# Chaperones in concert: Orchestrating co-translational protein folding in the cell

**DOI:** 10.1016/j.molcel.2024.06.018

**Published:** 2024-07-11

**Authors:** Bob Schiffrin, Antonio N. Calabrese

**Affiliations:** 1 Astbury Centre for Structural Molecular Biology, School of Molecular and Cellular Biology, Faculty of Biological Sciences, University of Leeds, Leeds LS2 9JT, UK

## Abstract

In this issue of *Molecular Cell*, Roeselová et al.^1^ provide insights into co-translational folding of a multidomain protein in bacteria, revealing how the chaperones Trigger Factor, DnaJ, and DnaK work together to facilitate the folding of nascent chains.

The astonishingly diverse activities of proteins underpin all molecular processes in living organisms, and the machineries for making these extraordinary molecules are conserved from bacteria to humans.^2^ In the age of AlphaFold and RoseTTAFold, the final folded (“native”) state of proteins and their complexes can now often be predicted with high confidence. However, ensuring that proteins adopt their native state is a Herculean task for cells, particularly for proteins with complex folds and/or multidomain architectures. Even after some ~4 billion years of evolution, around a third of proteins still fail to reach their native states and are degraded.^3^

The blueprint for a protein’s final structure is entirely encoded in its amino acid sequence, but many proteins require additional cellular factors for correct folding. Key to this process are molecular chaperones that bind proteins in their non-native states to assist folding.^4^ Chaperones act on proteins as they emerge from the ribosome exit tunnel during synthesis, binding the growing polypeptide “nascent chain” to facilitate folding before protein synthesis is complete (“co-translational folding”). In bacteria, the major chaperones involved in co-translational folding are Trigger Factor (TF), DnaJ, and DnaK (Hsp40 and Hsp70 in humans, respectively).

Most protein-folding studies to date have focused on the folding of single-domain proteins in the absence of folding factors. While these studies have revealed important insights into folding processes, much remains to be discovered on how proteins are folded in a cellular context, especially how folding is aided by chaperones co-translationally.^5^ There are key questions remaining: when do different nascent chains start to fold? How are nascent chains recognized by different chaperones? Where are the binding sites on chaperones for nascent chains, and what are their specificities for different polypeptide regions? Do different chaperones recognize the “folded-ness” of nascent chains? And crucially, how do the repertoire of chaperones coordinate during co-translational folding?

In this issue, Roeselová et al.^1^ address these questions with a tour-de-force study deploying biochemistry and state-of-the-art structural proteomics (crosslinking-mass spectrometry [XL-MS], and hydrogen-deuterium exchange-mass spectrometry [HDX-MS]). They selected a ~1,000 amino acid substrate (β-galactosidase [β-gal]), consisting of five separate domains and arrested its folding at many different points during its translation, then purified the resulting complexes of ribosomes, nascent chains, and chaperones for characterization.

The authors provide a wealth of new information on co-translational folding in bacteria and confirm many results from previous studies that used isolated components. The data show that chaperones are excluded early in translation, providing a window for nascent-chain-modifying enzymes and favoring partial folding before chaperone interaction. Proximity to the ribosome likely prevents aggregation of short nascent chains, reducing the need for chaperones at this stage. The chaperone TF acts as the “first responder” as nascent chains emerge from the ribosome, waiting at the ribosomal exit tunnel. Using elegant domain duplication experiments, the authors demonstrate that chaperone exclusion from early nascent chains is achieved by the TF ribosome binding domain (RBD) acting as a “molecular ruler.” This gives time and room for nascent chains to explore conformational space and/or engage with nascent-chain-modifying enzymes prior to TF interactions, mediated by both hydrophobic and electrostatic interactions. Consistent with this, the authors found that TF recognizes compact folding intermediates that presumably form prior to chaperone binding.

TF prevents early DnaJ/K binding (up to ~250 residues) by efficiently competing with DnaJ for nascent-chain binding. DnaJ binds the β-gal nascent chain using a large surface area, and not exclusively via hydrophobic interactions, similarly to TF, allowing it to recognize a variety of folded states. The authors show that DnaJ is critically important for the recruitment of DnaK and provide evidence that DnaK interactions are dictated by co-translational folding, as DnaK binding stoichiometry does not increase with nascent-chain length despite the creation of more predicted binding sites as synthesis proceeds. The fact that TF ensures that DnaJ/K can access only N-terminal domains that have escaped ribosome-bound TF, together with the observation that TF can fold proteins while bound,^6^ suggests that in the cell, DnaJ/K predominantly encounters substantially folded chains. Overall, the length and folding state of nascent chains appear to dictate the timing and mechanism of chaperone engagement (Figure 1).

Continuous chaperone engagement likely prevents inter-domain misfolding during synthesis for large, complex proteins. Here, the authors have focused on TF, DnaJ, and DnaK, which act early during protein biogenesis, and their data suggest that these chaperones act to stabilize structured states. For β-gal, it appears that partial folding of nascent chains is not reversed by chaperone binding, providing evidence that chaperone binding does not compete with co-translational folding. However, it remains unclear if/how structural remodeling and “rescuing” of misfolded states occur while ribosome-bound and how this is coordinated with the other major chaperone system in *E. coli*, GroEL/S. How chaperones facilitate biogenesis of proteins that form homo- or hetero-oligo-meric assemblies also remains an open question. For example, chaperones may preferentially bind to protein regions that are buried in the native oligomeric state, as these could comprise surfaces that promote aggregation.

Whether the findings in Roeselová et al. apply universally to co-translational protein folding across the Tree of Life remains to be elucidated. In eukaroytes, the protein-folding problem is more complex than prokaryotes due to larger proteomes and more individual domains per protein.^2^ The proteostasis network has thus evolved to be more complex; in place of TF, there are two eukaryotic ribosome-associated chaperone systems, the heterodimeric nascent polypeptide-associated complex (NAC) and the ribosome-associated complex (RAC).^7^ Un-raveling the precise mechanism of action of each of these ribosome-associated chaperone complexes, their interaction with chaperones and co-chaperones in the cell, and how together they can nurture the co-translational folding of complex eukaryotic proteins will require detailed investigation.

In summary, Roeselová et al.^1^ have exploited XL-MS and HDX-MS to generate a wealth of insights into co-translational folding of a multidomain protein. Understanding the folding of these complex proteins in the cell is crucial since duplication and shuffling events within genomes have resulted in the majority of proteins in all proteomes consisting of multiple domains,^8^ made from the rather limited set of single-domain protein folds used in nature (~1,500^9^). This study has revealed specific binding regions and chaperone interactions at different stages of protein synthesis. Importantly, the authors demonstrate that it is now possible, using biochemical reconstitution and state-of-the-art mass spectrometry methodologies, to map in molecular detail the coordinated, sequential interactions between chaperones and nascent chains during synthesis. This opens the door to exciting new discoveries in the co-translational folding field, including potential biotechnological applications in developing protein-expression systems for difficult-to-fold proteins and a better understanding of disease-causing mutations that promote toxic misfolded protein species. Understanding exactly how proteins reach their native states in the complex, crowded environments found in cells is poorly understood at the molecular level. This work has set the stage for many more exciting insights into how sequential, coordinated chaperone action during protein synthesis assists in maintaining a healthy cellular proteome.

## Figures and Tables

**Figure 1:**
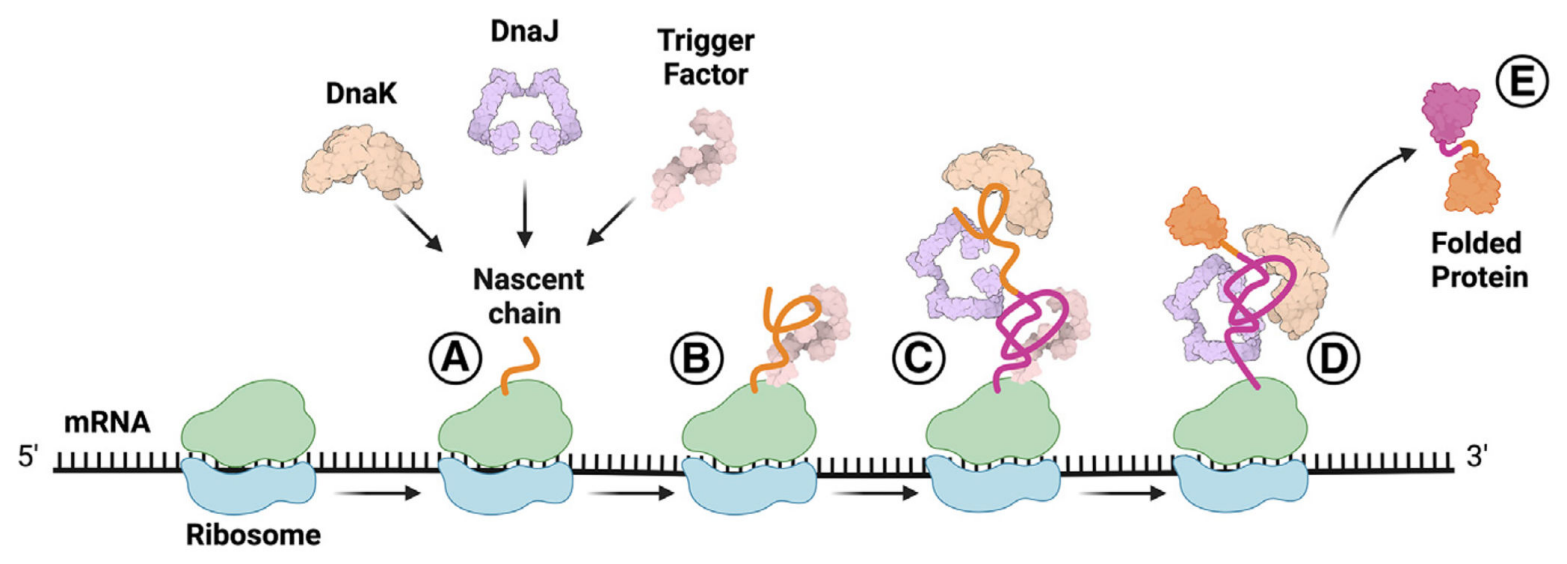
Model for spatial and temporal coordination between chaperones during assisted co-translational protein folding in bacteria (A) Short nascent chains emerging from the ribosome are excluded from chaperone binding. (B) Once a nascent chain > 100 residues emerges from the ribosome, Trigger Factor (TF) engages the nascent chain to protect it from misfolding and aggregation. (C) As protein synthesis continues, exemplified here for a two-domain protein (represented by one orange and one purple domain), the N-terminal domain is passed to DnaJ/DnaK, while the C-terminal domain is engaged by TF. (D) The N-terminal domain is then able to fold, and as protein synthesis continues, the C-terminal domain can be engaged by DnaJ/DnaK to assist folding. (E) The folded protein is released from the ribosome.

## References

[1] Roeselová A, Maslen SL, Shivakumaraswamy S, Pellowe GA, Howell S, Joshi D, Redmond J, Kjær S, Skehel M, Balchin D (2024). Mechanism of chaperone coordination during cotranslational protein folding in bacteria. Mol Cell.

[2] Rebeaud ME, Mallik S, Goloubinoff P, Tawfik DS (2021). On the evolution of chaperones and cochaperones and the expansion of proteomes across the Tree of Life. Proc Natl Acad Sci USA.

[3] Schubert U, Antón LC, Gibbs J, Norbury CC, Yewdell JW, Bennink JR (2000). Rapid degradation of a large fraction of newly synthesized proteins by proteasomes. Nature.

[4] Hartl FU, Bracher A, Hayer-Hartl M (2011). Molecular chaperones in protein folding and proteostasis. Nature.

[5] Bartlett AI, Radford SE (2009). An expanding arsenal of experimental methods yields an explosion of insights into protein folding mechanisms. Nat Struct Mol Biol.

[6] Wu K, Minshull TC, Radford SE, Calabrese AN, Bardwell JCA (2022). Trigger factor both holds and folds its client proteins. Nat Commun.

[7] Kramer G, Shiber A, Bukau B (2019). Mechanisms of Cotranslational Maturation of Newly Synthesized Proteins. Annu Rev Biochem.

[8] Han JH, Batey S, Nickson AA, Teichmann SA, Clarke J (2007). The folding and evolution of multidomain proteins. Nat Rev Mol Cell Biol.

[9] Andreeva A, Kulesha E, Gough J, Murzin AG (2020). The SCOP database in 2020: expanded classification of representative family and superfamily domains of known protein structures. Nucleic Acids Res.

